# [(*Z*)-Isopropoxy(4-nitrophenylimino)methanethiolato-κ*S*](tricyclo­hexyl­phosphine-κ*P*)gold(I)

**DOI:** 10.1107/S1600536809042767

**Published:** 2009-10-23

**Authors:** Soo Yei Ho, Edward R. T. Tiekink

**Affiliations:** a Department of Chemistry, National University of Singapore, Singapore 117543; b Department of Chemistry, University of Malaya, 50603 Kuala Lumpur, Malaysia

## Abstract

In the title compound, [Au(C_10_H_11_N_2_O_3_S)(C_18_H_33_P)], the gold(I) atom is linearly coordinated within a SP donor set. The distortion from linearity [S—Au—P = 177.54 (3)°] can be traced to an intra­molecular Au⋯O contact of 3.009 (3) Å. In the crystal, layers of mol­ecules are stabilized by a combination of C—H⋯O and C—H⋯π inter­actions.

## Related literature

For structural systematics and luminescence properties of phosphinegold(I) carbonimidothio­ates, see: Ho *et al.* (2006 ▶); Ho & Tiekink (2007 ▶); Kuan *et al.* (2008 ▶). For the synthesis, see Hall *et al.* (1993 ▶).
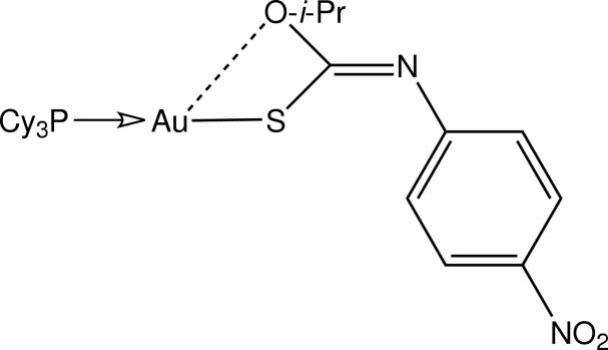


## Experimental

### 

#### Crystal data

[Au(C_10_H_11_N_2_O_3_S)(C_18_H_33_P)]
*M* *_r_* = 716.65
Triclinic, 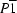

*a* = 9.0965 (3) Å
*b* = 13.1025 (4) Å
*c* = 13.2541 (5) Å
α = 80.030 (1)°
β = 75.170 (2)°
γ = 89.215 (1)°
*V* = 1503.26 (9) Å^3^
*Z* = 2
Mo *K*α radiation
μ = 5.05 mm^−1^
*T* = 223 K
0.36 × 0.26 × 0.05 mm

#### Data collection

Bruker SMART CCD diffractometer
Absorption correction: multi-scan (*SADABS*; Bruker, 2000 ▶) *T*_min_ = 0.508, *T*_max_ = 1
10654 measured reflections
6851 independent reflections
6092 reflections with *I* > 2σ(*I*)
*R*_int_ = 0.030

#### Refinement

*R*[*F* ^2^ > 2σ(*F* ^2^)] = 0.031
*wR*(*F* ^2^) = 0.072
*S* = 1.00
6851 reflections
325 parameters
H-atom parameters constrained
Δρ_max_ = 1.33 e Å^−3^
Δρ_min_ = −1.04 e Å^−3^

### 

Data collection: *SMART* (Bruker, 2000 ▶); cell refinement: *SAINT* (Bruker, 2000 ▶); data reduction: *SAINT*; program(s) used to solve structure: *PATTY* in *DIRDIF92* (Beurskens *et al.*, 1992 ▶); program(s) used to refine structure: *SHELXL97* (Sheldrick, 2008 ▶); molecular graphics: *ORTEP-3* (Farrugia, 1997 ▶) and *DIAMOND* (Brandenburg, 2006 ▶); software used to prepare material for publication: *SHELXL97*.

## Supplementary Material

Crystal structure: contains datablocks global, I. DOI: 10.1107/S1600536809042767/hb5149sup1.cif

Structure factors: contains datablocks I. DOI: 10.1107/S1600536809042767/hb5149Isup2.hkl

Additional supplementary materials:  crystallographic information; 3D view; checkCIF report

## Figures and Tables

**Table 1:** Selected geometric parameters (Å, °)

Au—P1	2.2655 (9)
Au—S1	2.3116 (9)

**Table 2:** Hydrogen-bond geometry (Å, °)

*D*—H⋯*A*	*D*—H	H⋯*A*	*D*⋯*A*	*D*—H⋯*A*
C17—H17⋯O3^i^	0.99	2.58	3.558 (6)	169
C27—H27b⋯*Cg*1^ii^	0.98	2.81	3.778 (5)	168

Symmetry codes: (i) 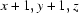; (ii) 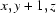. *Cg*1 is the centroid of the C2–C7 ring.

**Fig. 1.**
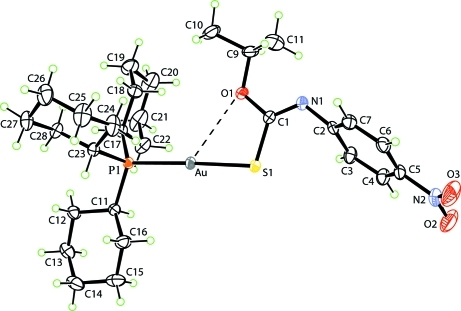
Molecular structure of (I) showing displacement ellipsoids at the 50% probability level.

**Fig. 2.**
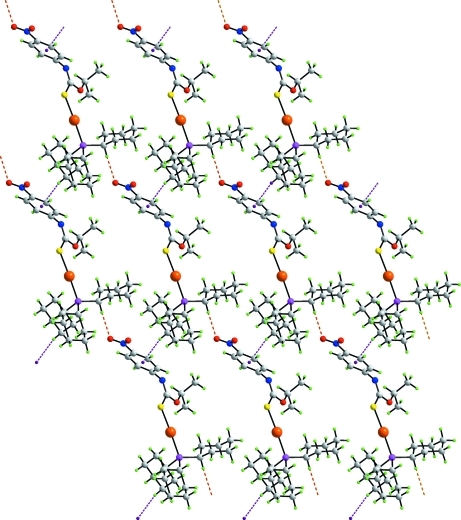
Supramolecular layer formation in (I) mediated by C—H···O and C—H···π contacts (orange and purple dashed lines, respectively). Colour code: Au, orange; S, yellow; P, pink; O, red; N, blue; C, grey; and H, green.

## supplementary crystallographic information

### Comment

The structure of the title compound, (I), was determined as a part of an on-going study of the structural systematics, including luminescence properties of molecules related to the general formula *R*_3_PAu[SC(OR')\d NR''] for *R*, *R*' and *R*'' = alkyl and aryl (Ho *et al.* 2006; Ho & Tiekink, 2007; Kuan *et al.*, 2008). The Au atoms in (I) exists in the expected linear geometry defined by S and P atoms (Au—S = 2.3116 (9) Å and Au—P = 2.2655 (9) Å), Fig. 1, with the deviation from the ideal 180° angle (S1—Au—P1 = 177.54 (3)°) being related to the close approach of the O1 atom, 3.009 (3) Å; this is the normally observed orientation of the carbonimidothioate anion when coordinated to phosphinegold(I) centres. The conformation about the C1N1 (1.275 (5) Å) is *Z* and the C1—S1 distance (1.739 (4) Å) indicates the ligand is functioning as a thiolate.

The formation of C—H···O and C—H···π interactions, Table 1, lead to supramolecular arrays in the *ab* plane, Fig. 2. These stack to consolidate the crystal packing.

### Experimental

Compound (I) was prepared following the standard literature procedure from the reaction of Cy_3_PAuCl and i-PrOC(S)N(H)C_6_H_4_NO_2_-4 in the presence of base (Hall *et al.*, 1993).  Yellow plates of (I) were obtained from the layering of ethanol on a dichloromethane solution of (I); m. pt. 434–436 K. Analysis for C_28_H_44_AuN_2_O_3_PS: found (calculated): C: 46.84 (46.93); H: 6.01 (6.19); N: 4.05 (3.91); S: 4.52 (4.47). IR (cm^-1^): ν(C—S) 1109 s, 875*m*; ν(C—N) 1590 s; ν(C—O) 1150*m*. ^31^P{^1^H} NMR: δ 56.8 p.p.m.

### Refinement

The H atoms were geometrically placed (C—H = 0.94–0.99 Å) and refined as riding with *U*_iso_(H) = 1.2*U*_eq_(C). The maximum and minimum residual electron density peaks of 1.33 and 1.04 e Å^-3^, respectively, were located 0.89 Å and 1.35 Å from the Au atom.

### Crystal data

**Table:**

[Au(C_10_H_11_N_2_O_3_S)(C_18_H_33_P)]	*Z* = 2
*M**_r_* = 716.65	*F*(000) = 720
Triclinic, *P*1	*D*_x_ = 1.583 Mg m^−^^3^
Hall symbol: -P 1	Mo *K*α radiation, λ = 0.71069 Å
*a* = 9.0965 (3) Å	Cell parameters from 5138 reflections
*b* = 13.1025 (4) Å	θ = 2.3–29.6°
*c* = 13.2541 (5) Å	µ = 5.05 mm^−^^1^
α = 80.030 (1)°	*T* = 223 K
β = 75.170 (2)°	Plate, yellow
γ = 89.215 (1)°	0.36 × 0.26 × 0.05 mm
*V* = 1503.26 (9) Å^3^	

### Data collection

**Table:**

Bruker SMART CCD diffractometer	6851 independent reflections
Radiation source: fine-focus sealed tube	6092 reflections with *I* > 2σ(*I*)
graphite	*R*_int_ = 0.030
ω scans	θ_max_ = 27.5°, θ_min_ = 1.6°
Absorption correction: multi-scan (*SADABS*; Bruker, 2000)	*h* = −11→11
*T*_min_ = 0.508, *T*_max_ = 1	*k* = −17→16
10654 measured reflections	*l* = −12→17

### Refinement

**Table:**

Refinement on *F*^2^	Primary atom site location: structure-invariant direct methods
Least-squares matrix: full	Secondary atom site location: difference Fourier map
*R*[*F*^2^ > 2σ(*F*^2^)] = 0.031	Hydrogen site location: inferred from neighbouring sites
*wR*(*F*^2^) = 0.072	H-atom parameters constrained
*S* = 1.00	*w* = 1/[σ^2^(*F*_o_^2^) + (0.0212*P*)^2^] where *P* = (*F*_o_^2^ + 2*F*_c_^2^)/3
6851 reflections	(Δ/σ)_max_ = 0.002
325 parameters	Δρ_max_ = 1.33 e Å^−^^3^
0 restraints	Δρ_min_ = −1.04 e Å^−^^3^

### Special details

**Table:**

Geometry. All s.u.'s (except the s.u. in the dihedral angle between two l.s. planes) are estimated using the full covariance matrix. The cell s.u.'s are taken into account individually in the estimation of s.u.'s in distances, angles and torsion angles; correlations between s.u.'s in cell parameters are only used when they are defined by crystal symmetry. An approximate (isotropic) treatment of cell s.u.'s is used for estimating s.u.'s involving l.s. planes.
Refinement. Refinement of *F*^2^ against ALL reflections. The weighted *R*-factor *wR* and goodness of fit *S* are based on *F*^2^, conventional *R*-factors *R* are based on *F*, with *F* set to zero for negative *F*^2^. The threshold expression of *F*^2^ > 2σ(*F*^2^) is used only for calculating *R*-factors(gt) *etc*. and is not relevant to the choice of reflections for refinement. *R*-factors based on *F*^2^ are statistically about twice as large as those based on *F*, and *R*- factors based on ALL data will be even larger.

### Fractional atomic coordinates and isotropic or equivalent isotropic displacement parameters (Å^2^)

**Table:**

	*x*	*y*	*z*	*U*_iso_*/*U*_eq_	
Au	0.773135 (15)	0.951953 (10)	0.686272 (11)	0.02645 (6)	
S1	0.67339 (12)	0.78988 (7)	0.68902 (8)	0.0338 (2)	
P1	0.86330 (10)	1.11387 (7)	0.68006 (7)	0.02323 (18)	
O1	0.8219 (3)	0.7604 (2)	0.8343 (2)	0.0354 (6)	
O2	0.3463 (5)	0.3639 (3)	0.5924 (3)	0.0756 (12)	
O3	0.1780 (5)	0.3537 (3)	0.7411 (3)	0.0799 (13)	
N1	0.6794 (4)	0.6193 (2)	0.8345 (3)	0.0347 (7)	
N2	0.3021 (5)	0.3830 (3)	0.6818 (3)	0.0503 (10)	
C1	0.7256 (4)	0.7124 (3)	0.7938 (3)	0.0290 (8)	
C2	0.5837 (4)	0.5653 (3)	0.7926 (3)	0.0282 (8)	
C3	0.6297 (5)	0.5421 (3)	0.6909 (3)	0.0366 (9)	
H3	0.7237	0.5686	0.6460	0.044*	
C4	0.5395 (5)	0.4810 (3)	0.6555 (3)	0.0383 (9)	
H4	0.5723	0.4643	0.5872	0.046*	
C5	0.4013 (5)	0.4446 (3)	0.7205 (3)	0.0333 (8)	
C6	0.3506 (5)	0.4670 (3)	0.8218 (3)	0.0363 (9)	
H6	0.2548	0.4421	0.8654	0.044*	
C7	0.4444 (5)	0.5269 (3)	0.8573 (3)	0.0344 (9)	
H7	0.4128	0.5417	0.9264	0.041*	
C8	0.8622 (5)	0.7039 (4)	0.9279 (3)	0.0417 (10)	
H8	0.7722	0.6629	0.9748	0.050*	
C9	0.9881 (7)	0.6324 (5)	0.8957 (5)	0.0675 (15)	
H9A	0.9539	0.5815	0.8608	0.101*	
H9B	1.0172	0.5973	0.9580	0.101*	
H9C	1.0749	0.6719	0.8472	0.101*	
C10	0.9037 (6)	0.7859 (4)	0.9832 (4)	0.0558 (13)	
H10A	0.8175	0.8297	1.0017	0.084*	
H10B	0.9895	0.8278	0.9365	0.084*	
H10C	0.9308	0.7532	1.0471	0.084*	
C11	0.8713 (4)	1.1952 (3)	0.5512 (3)	0.0278 (8)	
H11	0.9457	1.1634	0.4979	0.033*	
C12	0.9286 (5)	1.3074 (3)	0.5386 (3)	0.0376 (9)	
H12A	0.8585	1.3429	0.5897	0.045*	
H12B	1.0288	1.3077	0.5534	0.045*	
C13	0.9401 (5)	1.3650 (3)	0.4259 (3)	0.0462 (11)	
H13A	0.9708	1.4376	0.4205	0.055*	
H13B	1.0188	1.3340	0.3758	0.055*	
C14	0.7909 (6)	1.3610 (4)	0.3959 (3)	0.0499 (12)	
H14A	0.8048	1.3945	0.3219	0.060*	
H14B	0.7149	1.3991	0.4405	0.060*	
C15	0.7342 (6)	1.2499 (4)	0.4098 (4)	0.0522 (12)	
H15A	0.8050	1.2140	0.3596	0.063*	
H15B	0.6349	1.2496	0.3937	0.063*	
C16	0.7194 (5)	1.1922 (4)	0.5219 (3)	0.0421 (10)	
H16A	0.6871	1.1200	0.5273	0.050*	
H16B	0.6416	1.2240	0.5718	0.050*	
C17	1.0588 (4)	1.1123 (3)	0.6950 (3)	0.0273 (7)	
H17	1.0901	1.1835	0.6983	0.033*	
C18	1.0682 (5)	1.0405 (4)	0.7969 (4)	0.0432 (10)	
H18A	1.0012	1.0648	0.8580	0.052*	
H18B	1.0335	0.9703	0.7959	0.052*	
C19	1.2311 (5)	1.0382 (4)	0.8079 (4)	0.0628 (17)	
H19A	1.2633	1.1075	0.8138	0.075*	
H19B	1.2355	0.9905	0.8727	0.075*	
C20	1.3384 (6)	1.0032 (5)	0.7126 (5)	0.077 (2)	
H20A	1.4430	1.0053	0.7195	0.092*	
H20B	1.3119	0.9315	0.7105	0.092*	
C21	1.3287 (5)	1.0722 (5)	0.6107 (5)	0.0682 (18)	
H21A	1.3943	1.0458	0.5504	0.082*	
H21B	1.3660	1.1423	0.6096	0.082*	
C22	1.1665 (4)	1.0770 (4)	0.5990 (4)	0.0429 (10)	
H22A	1.1327	1.0083	0.5923	0.052*	
H22B	1.1635	1.1255	0.5344	0.052*	
C23	0.7423 (4)	1.1798 (3)	0.7827 (3)	0.0289 (8)	
H23	0.6684	1.2184	0.7485	0.035*	
C24	0.6475 (5)	1.1041 (4)	0.8751 (4)	0.0501 (12)	
H24A	0.7151	1.0603	0.9096	0.060*	
H24B	0.5865	1.0590	0.8486	0.060*	
C25	0.5415 (6)	1.1597 (4)	0.9567 (4)	0.0581 (14)	
H25A	0.4642	1.1954	0.9252	0.070*	
H25B	0.4892	1.1083	1.0180	0.070*	
C26	0.6273 (7)	1.2368 (4)	0.9930 (4)	0.0589 (14)	
H26A	0.6969	1.2004	1.0313	0.071*	
H26B	0.5555	1.2736	1.0418	0.071*	
C27	0.7166 (5)	1.3140 (3)	0.9004 (4)	0.0456 (11)	
H27A	0.7749	1.3614	0.9262	0.055*	
H27B	0.6459	1.3551	0.8665	0.055*	
C28	0.8249 (4)	1.2610 (3)	0.8189 (3)	0.0333 (8)	
H28A	0.8742	1.3132	0.7575	0.040*	
H28B	0.9044	1.2280	0.8499	0.040*	

### Atomic displacement parameters (Å^2^)

**Table:**

	*U*^11^	*U*^22^	*U*^33^	*U*^12^	*U*^13^	*U*^23^
Au	0.02825 (8)	0.02106 (8)	0.03289 (9)	−0.00194 (5)	−0.01194 (6)	−0.00612 (6)
S1	0.0446 (6)	0.0230 (5)	0.0409 (5)	−0.0047 (4)	−0.0243 (5)	−0.0045 (4)
P1	0.0221 (4)	0.0206 (4)	0.0284 (4)	−0.0012 (3)	−0.0088 (4)	−0.0046 (4)
O1	0.0460 (17)	0.0296 (15)	0.0351 (14)	−0.0112 (12)	−0.0207 (13)	−0.0016 (12)
O2	0.096 (3)	0.075 (3)	0.065 (2)	−0.028 (2)	−0.024 (2)	−0.030 (2)
O3	0.068 (3)	0.089 (3)	0.081 (3)	−0.049 (2)	−0.012 (2)	−0.019 (2)
N1	0.045 (2)	0.0258 (17)	0.0353 (18)	−0.0082 (14)	−0.0147 (15)	−0.0026 (14)
N2	0.058 (3)	0.033 (2)	0.061 (3)	−0.0156 (18)	−0.022 (2)	−0.0011 (19)
C1	0.032 (2)	0.0285 (19)	0.0291 (18)	−0.0006 (15)	−0.0101 (15)	−0.0085 (15)
C2	0.034 (2)	0.0182 (17)	0.0319 (19)	−0.0004 (14)	−0.0105 (16)	−0.0006 (14)
C3	0.031 (2)	0.034 (2)	0.041 (2)	−0.0088 (17)	−0.0006 (17)	−0.0101 (18)
C4	0.042 (2)	0.034 (2)	0.038 (2)	−0.0010 (18)	−0.0057 (18)	−0.0118 (18)
C5	0.037 (2)	0.0215 (19)	0.043 (2)	−0.0033 (16)	−0.0150 (18)	−0.0032 (16)
C6	0.035 (2)	0.031 (2)	0.038 (2)	−0.0079 (17)	−0.0078 (17)	0.0016 (17)
C7	0.038 (2)	0.033 (2)	0.0283 (19)	−0.0026 (17)	−0.0027 (16)	−0.0038 (16)
C8	0.050 (3)	0.049 (3)	0.028 (2)	−0.011 (2)	−0.0199 (19)	0.0050 (18)
C9	0.076 (4)	0.063 (4)	0.070 (4)	0.017 (3)	−0.037 (3)	−0.005 (3)
C10	0.067 (3)	0.065 (3)	0.045 (3)	−0.012 (3)	−0.030 (2)	−0.013 (2)
C11	0.0291 (19)	0.0265 (19)	0.0289 (18)	−0.0001 (15)	−0.0096 (15)	−0.0042 (15)
C12	0.043 (2)	0.030 (2)	0.040 (2)	−0.0059 (17)	−0.0148 (19)	0.0032 (17)
C13	0.051 (3)	0.035 (2)	0.046 (2)	0.001 (2)	−0.009 (2)	0.004 (2)
C14	0.062 (3)	0.051 (3)	0.032 (2)	0.024 (2)	−0.009 (2)	−0.001 (2)
C15	0.052 (3)	0.067 (3)	0.045 (3)	0.012 (2)	−0.028 (2)	−0.005 (2)
C16	0.041 (2)	0.047 (3)	0.042 (2)	−0.002 (2)	−0.021 (2)	−0.004 (2)
C17	0.0212 (17)	0.0267 (18)	0.038 (2)	0.0028 (14)	−0.0116 (15)	−0.0118 (16)
C18	0.046 (3)	0.040 (2)	0.051 (3)	0.0103 (19)	−0.025 (2)	−0.009 (2)
C19	0.060 (4)	0.069 (4)	0.084 (4)	0.031 (3)	−0.053 (3)	−0.033 (3)
C20	0.047 (3)	0.089 (4)	0.128 (5)	0.040 (3)	−0.056 (3)	−0.066 (4)
C21	0.024 (2)	0.097 (5)	0.100 (4)	0.016 (2)	−0.014 (3)	−0.062 (4)
C22	0.027 (2)	0.059 (3)	0.049 (2)	0.0071 (19)	−0.0097 (18)	−0.026 (2)
C23	0.0272 (19)	0.0277 (19)	0.0325 (19)	0.0010 (15)	−0.0063 (15)	−0.0096 (15)
C24	0.048 (3)	0.042 (3)	0.049 (3)	−0.014 (2)	0.012 (2)	−0.014 (2)
C25	0.050 (3)	0.058 (3)	0.052 (3)	−0.010 (2)	0.019 (2)	−0.018 (2)
C26	0.080 (4)	0.046 (3)	0.041 (3)	0.004 (3)	0.009 (2)	−0.018 (2)
C27	0.048 (3)	0.033 (2)	0.055 (3)	0.0039 (19)	−0.005 (2)	−0.018 (2)
C28	0.030 (2)	0.035 (2)	0.037 (2)	−0.0027 (16)	−0.0066 (16)	−0.0150 (17)

### Geometric parameters (Å, °)

**Table:**

Au—P1	2.2655 (9)	C14—H14A	0.9800
Au—S1	2.3116 (9)	C14—H14B	0.9800
S1—C1	1.739 (4)	C15—C16	1.520 (6)
P1—C11	1.835 (4)	C15—H15A	0.9800
P1—C17	1.839 (3)	C15—H15B	0.9800
P1—C23	1.850 (4)	C16—H16A	0.9800
O1—C1	1.350 (4)	C16—H16B	0.9800
O1—C8	1.459 (5)	C17—C18	1.527 (6)
O2—N2	1.219 (5)	C17—C22	1.532 (5)
O3—N2	1.222 (5)	C17—H17	0.9900
N1—C1	1.275 (5)	C18—C19	1.525 (6)
N1—C2	1.403 (5)	C18—H18A	0.9800
N2—C5	1.459 (5)	C18—H18B	0.9800
C2—C7	1.380 (5)	C19—C20	1.522 (7)
C2—C3	1.390 (5)	C19—H19A	0.9800
C3—C4	1.371 (5)	C19—H19B	0.9800
C3—H3	0.9400	C20—C21	1.512 (9)
C4—C5	1.368 (6)	C20—H20A	0.9800
C4—H4	0.9400	C20—H20B	0.9800
C5—C6	1.384 (6)	C21—C22	1.522 (6)
C6—C7	1.386 (5)	C21—H21A	0.9800
C6—H6	0.9400	C21—H21B	0.9800
C7—H7	0.9400	C22—H22A	0.9800
C8—C9	1.493 (7)	C22—H22B	0.9800
C8—C10	1.504 (6)	C23—C24	1.513 (6)
C8—H8	0.9900	C23—C28	1.521 (5)
C9—H9A	0.9700	C23—H23	0.9900
C9—H9B	0.9700	C24—C25	1.533 (6)
C9—H9C	0.9700	C24—H24A	0.9800
C10—H10A	0.9700	C24—H24B	0.9800
C10—H10B	0.9700	C25—C26	1.497 (7)
C10—H10C	0.9700	C25—H25A	0.9800
C11—C16	1.531 (5)	C25—H25B	0.9800
C11—C12	1.534 (5)	C26—C27	1.506 (7)
C11—H11	0.9900	C26—H26A	0.9800
C12—C13	1.531 (6)	C26—H26B	0.9800
C12—H12A	0.9800	C27—C28	1.521 (5)
C12—H12B	0.9800	C27—H27A	0.9800
C13—C14	1.513 (6)	C27—H27B	0.9800
C13—H13A	0.9800	C28—H28A	0.9800
C13—H13B	0.9800	C28—H28B	0.9800
C14—C15	1.516 (7)		
			
P1—Au—S1	177.54 (3)	C16—C15—H15B	109.3
C1—S1—Au	103.52 (12)	H15A—C15—H15B	107.9
C11—P1—C17	106.11 (17)	C15—C16—C11	110.6 (4)
C11—P1—C23	107.07 (17)	C15—C16—H16A	109.5
C17—P1—C23	108.78 (16)	C11—C16—H16A	109.5
C11—P1—Au	110.76 (12)	C15—C16—H16B	109.5
C17—P1—Au	111.51 (12)	C11—C16—H16B	109.5
C23—P1—Au	112.32 (12)	H16A—C16—H16B	108.1
C1—O1—C8	117.5 (3)	C18—C17—C22	110.1 (3)
C1—N1—C2	121.7 (3)	C18—C17—P1	110.8 (3)
O2—N2—O3	123.4 (4)	C22—C17—P1	109.6 (2)
O2—N2—C5	118.7 (4)	C18—C17—H17	108.8
O3—N2—C5	117.9 (4)	C22—C17—H17	108.8
N1—C1—O1	119.8 (3)	P1—C17—H17	108.8
N1—C1—S1	126.3 (3)	C19—C18—C17	110.6 (4)
O1—C1—S1	113.8 (3)	C19—C18—H18A	109.5
C7—C2—C3	119.0 (3)	C17—C18—H18A	109.5
C7—C2—N1	118.7 (3)	C19—C18—H18B	109.5
C3—C2—N1	122.0 (4)	C17—C18—H18B	109.5
C4—C3—C2	120.8 (4)	H18A—C18—H18B	108.1
C4—C3—H3	119.6	C20—C19—C18	110.7 (4)
C2—C3—H3	119.6	C20—C19—H19A	109.5
C5—C4—C3	119.3 (4)	C18—C19—H19A	109.5
C5—C4—H4	120.4	C20—C19—H19B	109.5
C3—C4—H4	120.4	C18—C19—H19B	109.5
C4—C5—C6	121.7 (4)	H19A—C19—H19B	108.1
C4—C5—N2	119.6 (4)	C21—C20—C19	110.8 (4)
C6—C5—N2	118.7 (4)	C21—C20—H20A	109.5
C5—C6—C7	118.4 (4)	C19—C20—H20A	109.5
C5—C6—H6	120.8	C21—C20—H20B	109.5
C7—C6—H6	120.8	C19—C20—H20B	109.5
C2—C7—C6	120.8 (4)	H20A—C20—H20B	108.1
C2—C7—H7	119.6	C20—C21—C22	111.7 (5)
C6—C7—H7	119.6	C20—C21—H21A	109.3
O1—C8—C9	110.0 (4)	C22—C21—H21A	109.3
O1—C8—C10	105.3 (4)	C20—C21—H21B	109.3
C9—C8—C10	113.7 (4)	C22—C21—H21B	109.3
O1—C8—H8	109.3	H21A—C21—H21B	107.9
C9—C8—H8	109.3	C21—C22—C17	110.8 (3)
C10—C8—H8	109.3	C21—C22—H22A	109.5
C8—C9—H9A	109.5	C17—C22—H22A	109.5
C8—C9—H9B	109.5	C21—C22—H22B	109.5
H9A—C9—H9B	109.5	C17—C22—H22B	109.5
C8—C9—H9C	109.5	H22A—C22—H22B	108.1
H9A—C9—H9C	109.5	C24—C23—C28	112.1 (3)
H9B—C9—H9C	109.5	C24—C23—P1	112.4 (3)
C8—C10—H10A	109.5	C28—C23—P1	115.0 (3)
C8—C10—H10B	109.5	C24—C23—H23	105.4
H10A—C10—H10B	109.5	C28—C23—H23	105.4
C8—C10—H10C	109.5	P1—C23—H23	105.4
H10A—C10—H10C	109.5	C23—C24—C25	111.9 (4)
H10B—C10—H10C	109.5	C23—C24—H24A	109.2
C16—C11—C12	110.8 (3)	C25—C24—H24A	109.2
C16—C11—P1	111.6 (3)	C23—C24—H24B	109.2
C12—C11—P1	115.3 (3)	C25—C24—H24B	109.2
C16—C11—H11	106.1	H24A—C24—H24B	107.9
C12—C11—H11	106.1	C26—C25—C24	111.6 (4)
P1—C11—H11	106.1	C26—C25—H25A	109.3
C13—C12—C11	110.3 (3)	C24—C25—H25A	109.3
C13—C12—H12A	109.6	C26—C25—H25B	109.3
C11—C12—H12A	109.6	C24—C25—H25B	109.3
C13—C12—H12B	109.6	H25A—C25—H25B	108.0
C11—C12—H12B	109.6	C25—C26—C27	111.1 (4)
H12A—C12—H12B	108.1	C25—C26—H26A	109.4
C14—C13—C12	112.0 (4)	C27—C26—H26A	109.4
C14—C13—H13A	109.2	C25—C26—H26B	109.4
C12—C13—H13A	109.2	C27—C26—H26B	109.4
C14—C13—H13B	109.2	H26A—C26—H26B	108.0
C12—C13—H13B	109.2	C26—C27—C28	111.9 (4)
H13A—C13—H13B	107.9	C26—C27—H27A	109.2
C13—C14—C15	110.9 (4)	C28—C27—H27A	109.2
C13—C14—H14A	109.5	C26—C27—H27B	109.2
C15—C14—H14A	109.5	C28—C27—H27B	109.2
C13—C14—H14B	109.5	H27A—C27—H27B	107.9
C15—C14—H14B	109.5	C23—C28—C27	111.6 (3)
H14A—C14—H14B	108.0	C23—C28—H28A	109.3
C14—C15—C16	111.7 (4)	C27—C28—H28A	109.3
C14—C15—H15A	109.3	C23—C28—H28B	109.3
C16—C15—H15A	109.3	C27—C28—H28B	109.3
C14—C15—H15B	109.3	H28A—C28—H28B	108.0
			
C2—N1—C1—O1	−177.7 (3)	C12—C13—C14—C15	−55.3 (5)
C2—N1—C1—S1	4.0 (6)	C13—C14—C15—C16	55.8 (5)
C8—O1—C1—N1	−3.2 (6)	C14—C15—C16—C11	−56.6 (5)
C8—O1—C1—S1	175.2 (3)	C12—C11—C16—C15	56.6 (5)
Au—S1—C1—N1	170.3 (3)	P1—C11—C16—C15	−173.5 (3)
Au—S1—C1—O1	−8.0 (3)	C11—P1—C17—C18	−176.2 (3)
C1—N1—C2—C7	−121.8 (4)	C23—P1—C17—C18	68.9 (3)
C1—N1—C2—C3	63.4 (5)	Au—P1—C17—C18	−55.5 (3)
C7—C2—C3—C4	−0.8 (6)	C11—P1—C17—C22	−54.5 (3)
N1—C2—C3—C4	173.9 (4)	C23—P1—C17—C22	−169.4 (3)
C2—C3—C4—C5	1.4 (6)	Au—P1—C17—C22	66.2 (3)
C3—C4—C5—C6	−0.6 (6)	C22—C17—C18—C19	57.6 (4)
C3—C4—C5—N2	177.6 (4)	P1—C17—C18—C19	179.0 (3)
O2—N2—C5—C4	1.2 (6)	C17—C18—C19—C20	−57.8 (6)
O3—N2—C5—C4	−178.3 (4)	C18—C19—C20—C21	56.5 (6)
O2—N2—C5—C6	179.4 (4)	C19—C20—C21—C22	−55.8 (6)
O3—N2—C5—C6	−0.1 (6)	C20—C21—C22—C17	55.8 (6)
C4—C5—C6—C7	−0.8 (6)	C18—C17—C22—C21	−56.3 (5)
N2—C5—C6—C7	−179.0 (4)	P1—C17—C22—C21	−178.5 (4)
C3—C2—C7—C6	−0.6 (6)	C11—P1—C23—C24	144.4 (3)
N1—C2—C7—C6	−175.5 (4)	C17—P1—C23—C24	−101.3 (3)
C5—C6—C7—C2	1.4 (6)	Au—P1—C23—C24	22.6 (3)
C1—O1—C8—C9	82.9 (5)	C11—P1—C23—C28	−85.7 (3)
C1—O1—C8—C10	−154.3 (4)	C17—P1—C23—C28	28.6 (3)
C17—P1—C11—C16	172.7 (3)	Au—P1—C23—C28	152.5 (3)
C23—P1—C11—C16	−71.2 (3)	C28—C23—C24—C25	51.7 (6)
Au—P1—C11—C16	51.6 (3)	P1—C23—C24—C25	−176.9 (4)
C17—P1—C11—C12	−59.7 (3)	C23—C24—C25—C26	−53.9 (6)
C23—P1—C11—C12	56.4 (3)	C24—C25—C26—C27	55.9 (6)
Au—P1—C11—C12	179.1 (3)	C25—C26—C27—C28	−56.4 (6)
C16—C11—C12—C13	−55.8 (5)	C24—C23—C28—C27	−51.9 (5)
P1—C11—C12—C13	176.3 (3)	P1—C23—C28—C27	178.1 (3)
C11—C12—C13—C14	55.5 (5)	C26—C27—C28—C23	54.2 (5)

### Hydrogen-bond geometry (Å, °)

**Table:**

*D*—H···*A*	*D*—H	H···*A*	*D*···*A*	*D*—H···*A*
C17—H17···O3^i^	0.99	2.58	3.558 (6)	169
C27—H27b···Cg1^ii^	0.98	2.81	3.778 (5)	168

Symmetry codes: (i) *x*+1, *y*+1, *z*; (ii) *x*, *y*+1, *z*.
